# Hypoxia due to intrapulmonary vascular dilatation in a toddler with a congenital portacaval shunt: case report

**DOI:** 10.1186/s12890-019-0788-8

**Published:** 2019-02-22

**Authors:** Mohammed T. Alsamri, Mohamed A. Hamdan, Mohamed Sulaiman, Hassib Narchi, Abdul-Kader Souid

**Affiliations:** 1 Departments of Pediatrics at Tawam Hospital, Alain City, United Arab Emirates; 2 KidsHeart Medical Center, Alain City, United Arab Emirates; 3 College of Medicine and Health Sciences - UAE University, Alain City, United Arab Emirates

**Keywords:** Cyanosis, Hypoxia, Portosystemic shunt, Venous malformation, Hepatopulmonary syndrome, Finger clubbing

## Abstract

**Background:**

The term hepatopulmonary syndrome typically applies to cyanosis that results from “*intrapulmonary vascular dilatation*” due to advanced liver disease. Similar findings may result from a congenital portosystemic shunt without liver disease. An adverse consequence of such shunts is intrapulmonary vascular dilatation, which affects the microvascular gas exchange units for oxygen.

**Case presentation:**

Here, we describe a toddler with chronic cyanosis, exercise intolerance, and finger clubbing due to a malformation shunt between the portal vein and the inferior vena cava. A transcatheter embolization of the shunt resulted in resolution of his findings.

**Conclusions:**

Congenital portosystemic shunts need to be considered in the differential diagnosis of cyanosis.

**Electronic supplementary material:**

The online version of this article (10.1186/s12890-019-0788-8) contains supplementary material, which is available to authorized users.

## Background

Cyanosis frequently results from a cardiac or pulmonary disease. Other rare causes include abnormal hemoglobin (e.g., methemoglobinemia) or hepatopulmonary syndrome. The latter entity may result from a hepatic parenchymal pathology or, more rarely, from a congenital portosystemic shunt that causes intrapulmonary vascular dilatation leading to hypoxia [1, 2]. A high index of suspicion is necessary to identify shunts between the portal vein and the inferior vena cava as a possible etiology of cyanosis.

Here, we describe a 2-year-old boy with chronic cyanosis due to a large portosystemic shunt between the portal vein and the inferior vena cava. This report aims to increase awareness of this rare entity and emphasize that embolization of the shunt is curative.

## Case presentation

**Fig. 1:**
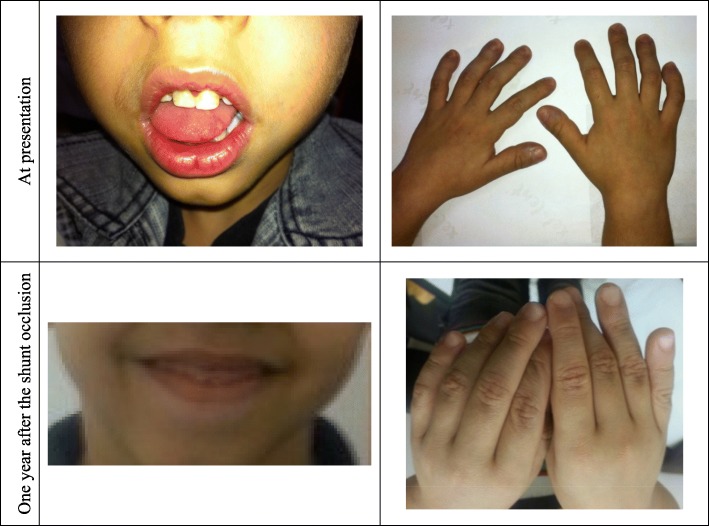
Resolution of cyanosis and digital clubbing after transcatheter occlusion of the portosystemic shunt

This 2-year-old toddler presented with a history of cyanosis for at least 4 months (Fig. 1). His oxygen saturation was 74 to 85% in room air. A purple discoloration in his lips and fingers was first noticed after mild crying or coughing episodes. Soon thereafter, the cyanosis was persistent and limited his activity. His antenatal echography showed mild cardiomegaly; otherwise, the pregnancy and delivery were uncomplicated. He was not taking any medication. The family history was unremarkable. A panel of investigations showed no evidence of cardiac, respiratory, immunologic, or gastrointestinal disease.

Physical examination showed normal growth and development. His respiratory rate was 32 breaths/min. His oxygen saturation was 85% in room air, and increased to 92% with 5 L/min oxygen by mask. There was no change in oxygen saturation with position (orthodeoxia). He had significant cyanosis and finger clubbing (Fig. 1). His chest wall, respiratory, cardiac, and abdominal examinations were normal.

Laboratory results showed normal sweat chloride concentration (23 mmol/L; normal, < 40), stool pancreatic elastase (382 μg/g; normal, > 200 μg/g), serum immunoglobulin, and peripheral lymphocyte immunophenotyping (Table 1). Screening for the most common 32 cystic fibrosis transmembrane conductance regulator (CFTR) mutations in our region was negative.

**Table 1:** Representative of his pre-operative laboratory results

	Results	Reference ranges
Arterial blood gas analysis^a^		
pH	7.42	7.35 to 7.45
Oxygen saturation	74%	95 to 99
PaO_2_	45 mmHg	93 to 100
PaCO_2_	29 mmHg	32 to 48
HCO_3_^−^	19 mmol/L	24 to 30
Methemoglobin	0.8%	< 1.0
Hemoglobin	127 g/L	105 to 127
Liver function		
Albumin	33 g/L	35 to 50
Total bilirubin	12.0 μmol/L	5.1 to 20.5
Direct bilirubin	3.0 μmol/L	1.70 to 8.60
Alkaline phosphatase	166 IU/L	32 to 91
Gamma-glutamyltranspeptidase	22 IU/L	7 to 50
Alanine aminotransferase	26 IU/L	17 to 63

^a^Room air while sitting

**Fig. 2:**
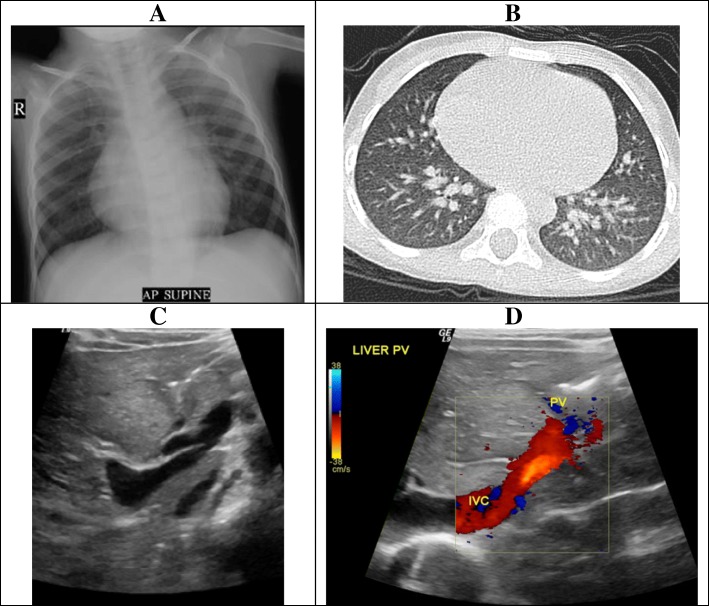
Representative of the pre-operative imaging. **a** Chest radiograph showing mild cardiomegaly with increased bronchovascular markings. **b** Contrast-enhanced axial chest computed tomography image showing normal lung parenchyma with a prominent pulmonary vasculature. **c** Ultrasound of the abdomen showing dilated inferior vena cava. **d** Doppler ultrasound of the abdomen showing portal vein (PV) shunting into the inferior vena cava (IVC)

His chest x-ray showed increased peripheral interstitial markings with mild cardiomegaly (Fig. 2). Computed tomography scan of the chest with contrast revealed normal lung parenchyma, prominent pulmonary vasculature (Fig. 2), and hepatic areas with different densities. Abdominal ultrasound showed dilated inferior vena cava associated with a portal-inferior vena cava high-flow fistula, with the portal system draining exclusively via the inferior vena cava (Fig. 2). Doppler study showed portal vein shunting into the inferior vena cava, as well as dilated branches of the portal and splenic veins (Fig. 2). A transthoracic echocardiography showed absence of structural cardiac defects, normal contractility, dilated (overloaded) left ventricle, and normal pulmonary pressures (the echocardiography showed mild tricuspid regurgitation, with a gradient of 22 mmHg indicating normal right ventricular pressure).

**Fig. 3:**
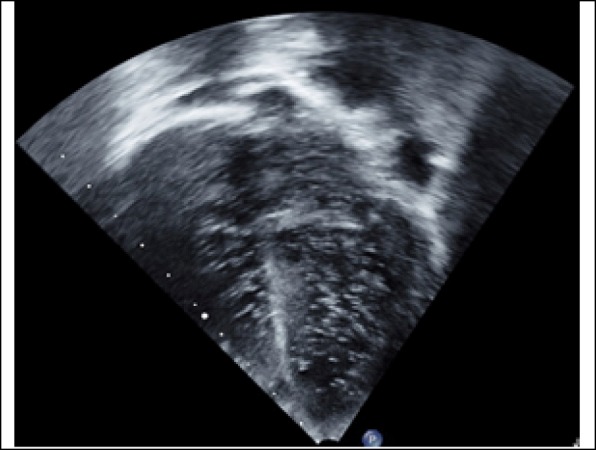
Trans-thoracic echocardiogram in four-chamber view with bubbles after injection of agitated saline. Opacification of the right atrium and right ventricle was noticed immediately. It is followed within one to two beats by complete opacification of the left atrium and left ventricle, indicating significant intrapulmonary shunting

A positive bubble contrast study was demonstrated by intravenous injection of 3.0 mL of agitated saline in the hand. Opacification of the right cardiac chambers was noticed immediately and followed within one to two beats by complete opacification of the left chambers, indicating an intrapulmonary shunt of at least 50% of the pulmonary blood flow (Fig. 3).

**Fig. 4:**
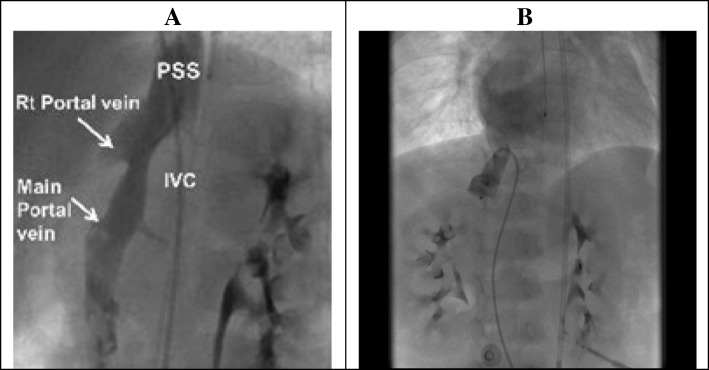
**a** Selective angiogram in the inferior vena cava (IVC). There was a connection between the portal vein and the inferior vena cava (IVC) through a portosystemic shunt (PSS) that measured about 25 mm in length and 11 mm in width. The right portal vein was hypoplastic. **b** Repeat angiogram in the inferior vena cava (IVC) after transcatheter embolization of the shunt with a vascular plug demonstrating the absence of residual flow

Cardiac catheterization showed significant intrapulmonary shunting. Angiography of the inferior vena cava and portal vein confirmed connection of the portal vein and the inferior vena cava through a portosystemic shunt that measured about 25 mm in length and 11 mm in width (Fig. 4a). The left portal vein had a normal caliber and the right portal vein was hypoplastic (Fig. 4a). A transcatheter embolization of the shunt was performed using a 16-mm Amplatzer Vascular Plug (AGA Medical Corporation, Golden Valley, Minnesota, USA), Fig. 4b. Complete occlusion of the shunt was confirmed by abdominal ultrasound, echocardiogram and angiogram. Two weeks after the procedure, his oxygen saturation was 95 to 100% in room air. Repeat contrast bubble study then showed absence of fistulous flow into the left atrium and left ventricle. His cyanosis and finger clubbing gradually disappeared (Fig. 1). He has been stable and asymptomatic since his last assessment 5 years after the procedure. Hemodynamic data from the cardiac catheterization are shown in the Additional files 1 and 2.

## Discussion

This brief report emphasizes the following points: (1) Portosystemic shunts (resulting in intrapulmonary vascular dilation) should be considered in the differential diagnosis of cyanosis; (2) Proper assessment of portosystemic shunts requires chest radiograph, echocardiogram with bubble test, and abdominal ultrasound with Doppler study; and (3) Closure of the shunt is curative.

Hepatopulmonary syndrome is easier to recognize in the context of severe liver disease. This toddler had cyanosis with no evidence of cardiac, pulmonary, or liver disease. The possibility of hepatopulmonary syndrome was considered after the finding of a portosystemic shunt on the abdominal ultrasound Doppler study. The findings on his chest radiograph, chest computerized tomography scan, and echocardiogram (bubble test) were consistent with intrapulmonary vascular dilatation as a cause of his hypoxia [1, 2]. Intrapulmonary vascular dilatations involve the pulmonary precapillary and capillary vessels that occur in the setting of portal hypertension or congenital portosystemic shunts [3, 4].

Contrast echocardiography is a useful diagnostic test for intrapulmonary vascular dilatation. This maneuver involves intravenous injection of microbubbles (> 10 μm in diameter) from agitated normal saline. Such bubbles are normally obstructed by the pulmonary capillaries (normal size, < 8 μm to 15 μm). In the presence of intrapulmonary vascular dilatation, these microbubbles rapidly transit the lung and appear in the left atrium within a few heart beats [1].

The bubble echocardiogram test showed opacification of the left heart chambers within 1–2 beats after its appearance in the right heart chambers. This finding raised the possibility of an intracardiac shunting, such as patent foramen ovale (PFO) [5]. However, his right heart pressure was normal. Furthermore, careful assessment with two-dimensional color flow mapping at very low velocity Doppler scale failed to demonstrate any intracardiac shunting. Unfortunately, the toddler did not cooperate with Valsalva maneuver, intended to raise the right atrial pressure and promote right to left shunting across the PFO.

**Fig. 5:**
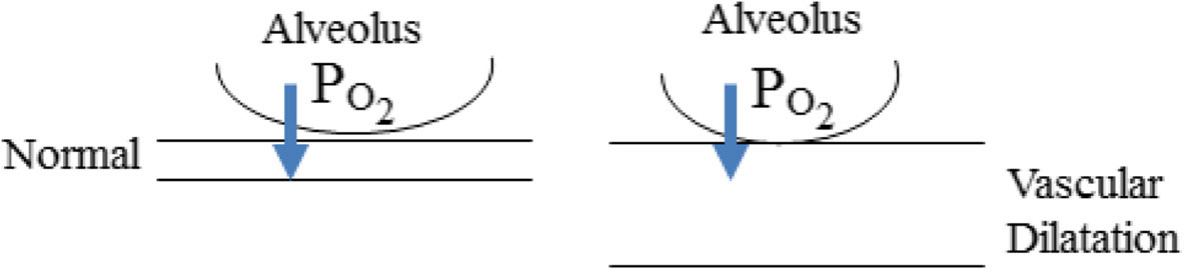
Schema depicting hypoxia in microvascular dilatation in the pulmonary arterial circulation

The mechanism of hypoxia in hepatopulmonary syndrome is microvascular dilatation within the pulmonary arterial circulation (Fig. 5) [6, 7]. This dilatation is caused by exposure to intestinal vasoactive mediators (e.g., nitric oxide) that bypassed the liver due to liver disease or congenital portosystemic shunt [4]. Consistently, exhaled nitric oxide, a potent pulmonary vasodilator, is elevated in patients with liver cirrhosis and hepatopulmonary syndrome [4]. Patients with liver cirrhosis have elevated plasma endothelin-1, which binds to its pulmonary endothelin-1B receptor and triggers the inducible and endothelial nitric oxide (iNOS and eNOS) synthases. Elevated nitric oxide causes pulmonary vasodilatation [4].

Intrapulmonary vascular dilatation is characterized by vessels diameters of 60 μm to 80 μm (normal, 8–15 μm). In dilated vessels, the distance for oxygen diffusion increases (Fig. 5). Oxygen diffuses more readily to red cells that are closer to the alveolar wall than those in the periphery (due to the shorter distance from the alveolar capillary membrane). Giving oxygen can overcome this diffusion disequilibrium within the alveolar gas exchange unit by increasing oxygen tension within the alveolus.

Due to vascular dilatation the volume of blood in the gas exchange units is increased, preventing full oxygen saturation per volume. This process worsens during exercise, cough or crying due to an even faster transit time of the red cells in the alveolar capillary unit. As previously suggested, hypoxemia may increase blood flow through intrapulmonary arteriovenous anastomoses [8]. Thus, the left heart contrast before the intervention in this patient may have been a result of the hypoxemia. Supplemental oxygen increases the driving pressure of oxygen and improves oxygenation, distinguishing this condition from hypoxia due to a right-to-left shunt [9].

Intrapulmonary vascular dilatation also leads to increased pulmonary blood flow, elevated perfusion ventilation mismatch, arteriovenous shunts, and increased cardiac output [10]. As intrapulmonary vascular dilatation often has a heterogeneous distribution in the lungs, the ventilation-perfusion mismatching may have a distinct positional component caused by redistribution of blood flow toward the lower lung zones. This phenomenon results in platypnea (decreased dyspnea when recumbent) and orthodeoxia (worsening of hypoxemia when setting up) [10].

In children, a preexisting liver disease is less common than congenital portosystemic shunts, which can be either extrahepatic or intrahepatic [11]. The complete absence of portal vein is called Abernethy malformation [11]. Type I- Abernethy malformation describes complete diversion of portal blood into the vena cava with an absence of the portal vein, which is consistent with the finding of hypoplastic portal vein in our patient with portal blood diverting into the vena cava through a side-to-side extrahepatic communication [11]. Spontaneous or interventional occlusion of the portosystemic shunts usually leads to complete resolution of the hepatopulmonary syndrome [2, 12].

Spontaneous closure is possible in children younger than 2 years, especially in intrahepatic shunts. Therefore, in these young children, therapeutic procedures may be postponed in the absence of symptoms or significant shunt ratio. Shunt closure procedures should be considered for symptomatic patients and those with shunts that persist beyond the first 2 years of life.

## Conclusions

Hepatopulmonary syndrome is a well-recognized complication of advanced liver diseases. Diagnosing this entity in in the absence of liver pathology requires a high index of suspicion. The presence of cyanosis and dyspnea without respiratory or cardiac pathology should lead to a careful evaluation of platypnea and orthodeoxia. In hepatopulmonary syndrome, the cyanosis improves with oxygen supplementation. Pulmonary images are useful to exclude lung pathology and may demonstrate the intrapulmonary vascular dilatation. Echocardiography is useful to exclude cyanotic heart disease; the study should also include the porto-caval area and the microbubble test. Angiography is required to establish the diagnosis of hepatopulmonary syndrome and congenital portosystemic shunt. Cyanosis and clubbing are reversible after shunt occlusion.

## Additional files

Additional file 1: Hemodynamic data from the cardiac catheterization. (DOCX 15 kb)

Additional file 2: Summary of the clinical course. (PDF 292 kb)

## Abbreviations

CFTR: Cystic fibrosis transmembrane conductance regulator
eNOS: Endothelial nitric oxide
eNOS: Endothelial nitric oxide synthase
iNOS: Inducible nitric oxide
IPAVA: Intrapulmonary arteriovenous anastomoses
IVC: Inferior vena cava
PV: Portal vein
SaO_2_: Arterial oxygen saturation
