# Supplementary material for: Hypoxia due to intrapulmonary vascular dilatation in a toddler with a congenital portacaval shunt: case report
Source: BMC Pulm Med. 2019 Feb 22;19:49. doi: 10.1186/s12890-019-0788-8 (PMC6387555; doi:10.1186/s12890-019-0788-8)
Supplement: Supplementary file 1 — Hemodynamic data from the cardiac catheterization. (DOCX 15 kb) [file 12890_2019_788_MOESM1_ESM.docx]

**Additional file 1**

**Hemodynamic data from the cardiac catheterization included:**

*On 35% O_2_:*

- *Saturation:* Saturation in the superior vena cava (SVC) was 84%, in the descending aorta (DAO) was 90%, and in the right pulmonary artery (RPA) was 78%. The cardiac output was normal. There was no intracardiac shunt, but significant intrapulmonary shunting was noted.
- *Pressure:* The right ventricular end-diastolic (RVED), right ventricular (RV), and right pulmonary artery (RPA) pressures were all normal. There was no right ventricular outflow tract obstruction or pulmonary stenosis. Left pulmonary artery (LPA) pressure and main pulmonary artery pressure (MPA) were 20/8 mm Hg (mean = 12). The right ventricular pressure was 20/6 mm Hg; right atrial pressure: a = 6 mm Hg, v = 2 mm Hg, mean = 4 mm Hg. Left ventricular end-diastolic (LVED), left ventricular (LV), and aortic pressures were all normal. There was no left ventricular outflow tract obstruction, aortic stenosis, or aortic coarctation. Left pulmonary capillary wedge (LPCW) pressure was: a = 7 mm Hg, v = 11 mm Hg, and mean = 10 mm Hg. LV pressure was 95/7 mm Hg, ascending aorta (AAO) and descending aorta (DAO) pressures were 90/47 mm Hg (mean = 61). Inferior vena cava (IVC) mean pressure was 9 mm Hg and mean portal vein pressure was 9 mm Hg.
- *Calculations:* Qp/Qs (pulmonary blood flow/ systemic blood flow) was 0.5 L/min/m^2^, Qs (systemic blood flow) was 15.4 L/min/m^2^, and Qp (pulmonary blood flow) was 7.7 L/min/m^2^. PVR (pulmonary vascular resistance) was 0.3 Woods units and SVR (systemic vascular resistance) was 4 Woods units.

*On 100% O_2_:*

- *Saturation:* Saturation in the superior vena cava (SVC) was 88% (PO_2_ = 66 mm Hg), in the descending aorta (DAO) was 100% (PO_2_ = 163 mm Hg), and in the left pulmonary artery (LPA) was 89% (PO_2_ = 68 mm Hg). There was no intracardiac shunt. Significant intrapulmonary shunting was noted, which decreased with 100% O_2_.
- *Pressure*: LPCW pressure was 10 mm Hg, LPA mean pressure was 12 mm Hg, DAO mean pressure was 60 mm Hg, and right atrial mean pressure was 6 mm Hg.
- Qp/Qs was 1.0, Qs = QP = 9.0 L/min/m^2^. PVR was 0.2 Woods units and SVR was 7.0 Woods units.
