# Supplementary material for: Hypoxia due to intrapulmonary vascular dilatation in a toddler with a congenital portacaval shunt: case report
Source: BMC Pulm Med. 2019 Feb 22;19:49. doi: 10.1186/s12890-019-0788-8 (PMC6387555; doi:10.1186/s12890-019-0788-8)
Supplement: Supplementary file 2 — Summary of the clinical course. (PDF 292 kb) [file 12890_2019_788_MOESM2_ESM.pdf]

Previously healthy 2 year old child, antenatal echography showed mild cardiomegaly

0

Current Illness: **Cyanosis** for 4- months

Physical Examination: Oxygen saturation 74% to 85% in room air: Finger clubbing

Diagnostic Evaluations:
Chest x-ray: increased peripheral interstitial markings with mild cardiomegaly

Abdominal ultrasound: dilated inferior vena cava, with portal vein shunting into the inferior vena cava

Echocardiography No structural cardiac defects, normal pulmonary pressures, positive bubble contrast study

Cardiac catheterization:
Significant intrapulmonary shunting
Portal vein- inferior vena cava portosystemic shunt

8-months

Transcatheter embolization of the shunt using a Vascular Plug **Complete occlusion** of the shunt was confirmed by abdominal ultrasound, echocardiogram and angiogram

2-weeks after closure

Two weeks after the procedure, his oxygen saturation was 95% to 100% in room air.

4-years follow up cyanosis and finger clubbing gradually disappeared
